# DNA methylation change of HIPK3 in Chinese rheumatoid arthritis and its effect on inflammation

**DOI:** 10.3389/fimmu.2022.1087279

**Published:** 2023-01-10

**Authors:** Ping Jiang, Kai Wei, Lingxia Xu, Cen Chang, Runrun Zhang, Jianan Zhao, Yehua Jin, Linshuai Xu, Yiming Shi, Yi Qian, Songtao Sun, Shicheng Guo, Rongsheng Wang, Yingying Qin, Dongyi He

**Affiliations:** ^1^ Guanghua Clinical Medical College, Shanghai University of Traditional Chinese Medicine, Shanghai, China; ^2^ Shanghai Guanghua Hospital of Integrated Traditional Chinese and Western Medicine, Shanghai, China; ^3^ Arthritis Institute of Integrated Traditional and Western medicine, Shanghai Chinese Medicine Research Institute, Shanghai, China; ^4^ The Second Affiliated Hospital of Shandong University of Traditional Chinese Medicine, Jinan, China; ^5^ Department of Medical Genetics, School of Medicine and Public Health, University of Wisconsin-Madison, Madison, WI, United States; ^6^ Computation and Informatics in Biology and Medicine, University of Wisconsin-Madison, Madison, WI, United States

**Keywords:** DNA methylation, rheumatoid arthritis, inflammation, HIPK3, methylation haplotypes

## Abstract

**Introduction:**

Homeodomain-interacting protein kinase 3 (HIPK3) plays an important role in cell proliferation, apoptosis, and inflammation. Over-expression of HIPK3 in immune cells in rheumatoid arthritis (RA) has been reported. In this study, we investigated blood methylation levels and clinical characteristics of RA in a Chinese population.

**Methods:**

A total of 235 patients with RA, 30 with osteoarthritis (OA), and 30 matched healthy controls were recruited. The methylation status of seven CpGs in the differentially methylated region of HIPK3 (cg05501357) was measured using targeted methylation-sequencing technology. The association between methylation haplotypes and the overall methylation status of HIPK3 with clinical characteristics was assessed using generalized linear regression.

**Results:**

All seven CpGs showed hypomethylation status in RA blood compared with OA and normal individuals (overall p= 1.143×10^-8^ and FDR= 2.799×10^-7^), which is consistent with the previously reported high expression of HIPK3 in RA immune cells. Among all seven CpGs, 33286785 showed the highest predictive power with an area under the curve (AUC) of 0.829; we received a higher AUC=0.864 when we combined HIPK3 with anti-citrullinated protein antibodies (ACPA -) and rheumatoid factor (RF +) in the prediction model, indicating that when a patient’s ACPA is negative, HIPK3 can assist RF as a new clinical index for the diagnosis of RA. We also found that HIPK3 methylation levels were negatively correlated with C-reactive protein (CRP; r= -0.16, p= 0.01). Methylation haplotypes were analyzed, and the full methylation haplotype (FMH; r= 0.16, p= 0.01) and full non-methylation haplotype (FNH; r= 0.18, p= 0.0061) were negatively correlated with CRP.

**Conclusion:**

Circulating blood methylation levels in the protein region of HIPK3 can be utilized as a supportive diagnostic biomarker and CRP level indicator for RA.

## Introduction

Rheumatoid arthritis (RA) is characterized by an abnormal or excessive inflammatory response. Under the action of certain disease factors, patients trigger an inflammatory response in the body and form a storm of inflammatory factors (1). Persistent inflammation can cause joint pain, progressive destruction, and joint dysfunction (2). In addition, inflammation may cause various complications, such as cardiovascular diseases (3). Therefore, effective control of inflammation is key to the treatment of RA, and can even be used as an indicator to evaluate the effectiveness of treatment. Doctors can also decide whether to adjust the treatment plan according to the progress of inflammation. It is important to study the specific molecular biological mechanisms of RA and identify inflammation-related biomarker.

Epigenetic mechanisms (4), such as deoxyribonucleic acid (DNA) methylation, participate in many important life activities (5), including cell differentiation and proliferation, organism aging (6), and tumorigenesis (7), and play an important role in the regulation of gene expression (8). In recent years, the potential role of DNA methylation in RA development has attracted the attention of researchers (9, 10). DNA methylation affects different aspects of RA and modulates epigenetic silencing of genes and cellular behavior, especially in fibroblast-like synoviocytes (FLS), whose abnormal proliferation promotes persistent inflammation and joint damage (11, 12). Thus, aberrant DNA methylation occurs in the pathogenesis of RA and contributes to its development.

Homeodomain-interacting protein kinase3 (HIPK3) is a group of conserved serine/threonine kinases that can regulate different transcription factors that to influence developmental processes, such as cell proliferation, differentiation, apoptosis and inflammatory responses (13). The conservation of HIPK3 is relatively low and it shares many functional domains with HIPK2 (14). A study found that the expression levels of HIPK3 mRNA and protein were significantly down-regulated in human non-small cell lung cancer (NSCLC) tissues, and HIPK3 silencing promoted the invasion and metastasis of NSCLC (15). Thus, HIPK3 may be a valuable biomarker for predicting the prognosis of patients with non-small cell lung cancer. In addition, HIPK3 may also be a novel kinase regulator of autophagy in Huntington disease (HD) cells, contributing to the accumulation of proteins and disease progression. Targeting HIPK3 may provide drug discovery opportunities for the treatment of HD and similar diseases (16).

Currently, there are few studies on HIPK3, particularly regarding its relationship with RA. In this study, we explored the relationship between HIPK3, methylation haplotypes in whole blood, and clinical indicators in patients with RA, especially the correlation between the inflammatory indicator CRP and erythrocyte sedimentation rate (ESR). The results may provide potential value for clinical applications to predict the degree of inflammation in patients with RA.

## Materials and methods

### Participants and peripheral blood collection

We recruited participants (235 RA patients, 30 with OA patients, and 30 healthy controls) between October 20 and November 30, 2021, from the Guanghua Hospital Precision Medicine Research Cohort [PMRC] (17). The inclusion criterion for RA was the American College of Rheumatology (ACR) 2010 criteria (18). The clinical data of all participants were recorded, and whole blood samples were collected. This study was approved by the Ethics Committee of the Guanghua Hospital (Approval No. 2018-K-12), and all participants provided written informed consent.

### DNA methylation detection for HIPK3

Genomic DNA was extracted from the peripheral blood of patients with RA, patients with OA, and healthy controls. Sample integrity: For agarose electrophoresis detection, the main strip was required to be clear, with no obvious dispersion and tailing. DNA was extracted using the EZ DNA Methylation-Gold Kit (ZYMO, CA, USA), as recommended by the manufacturer. Subsequently, a single PCR step was used to amplify the target region of the transformed sample. The primer sequences for HIPK3 were as follow that primer F, ATTTTGTTTTGATTTTGTGGTAGTTGTT; and primer R, CAAAAATCATAACAACTCAAACACAAC. Then, dilute the mixed single PCR products 10-20 times and specific tag sequences were added. Using primers with an index sequence, a specific tag sequence compatible with the illumina platform was introduced at the end of the library *via* PCR amplification. Finally, FastQ data were obtained by high-throughput sequencing using the 2×150 bp double-terminal sequencing mode on an Illumina Hiseq (Illumina, CA, USA).

### Statistical analyses

Spearman’s rank correlation analysis was used to assess the association between DNA methylation level and clinical data of patients with RA. Using One-Way ANOVA test to calculate the P values in baseline analysis. Using Kruskal-Wallis rank sum test to analysis the methylation difference level between RA, OA and healthy groups. Clinical significance of HIPK3 methylation level analyzed by receiver operating characteristic curve (ROC curve). All statistical analyses were performed by the IBM SPSS version 20.0 software (IBM Inc, Armonk, New York, USA), GraphPad Prism software (version 9.0), and Sangerbox (19).

## Results

### Basic information of participants

The study included 235 patients with RA, 30 patients with OA, and 30 healthy individuals, who were divided into RA, OA, and healthy groups. In the RA group, 203 cases were RF (+), accounting for 86.38% of the total, and 213 cases were ACPA (+), accounting for 90.64%. In baseline analysis, using One-Way ANOVA test to calculate the P values showed that there were no significant differences in age (p= 0.460), height (p= 0.376), and weight (p= 0.525) among the three groups, but there were significant statistical differences in erythrocyte sedimentation rate (ESR; p= 0.000) and C-reactive protein (CRP; p= 0.000) (**Supplement Table 1**). The levels of CRP and ESR are important indicators for the diagnosis of OA and RA, and the degree of inflammatory activity. The increase of ESR and CRP levels in patients with active OA and RA can not only aggravate joint swelling, but also indirectly reflect the degree of bone, joint and organ damage, which is one of the important indicators for the diagnosis of OA and RA.

### Differences in methylation level and correlation of HIPK3

**Figure 1:**
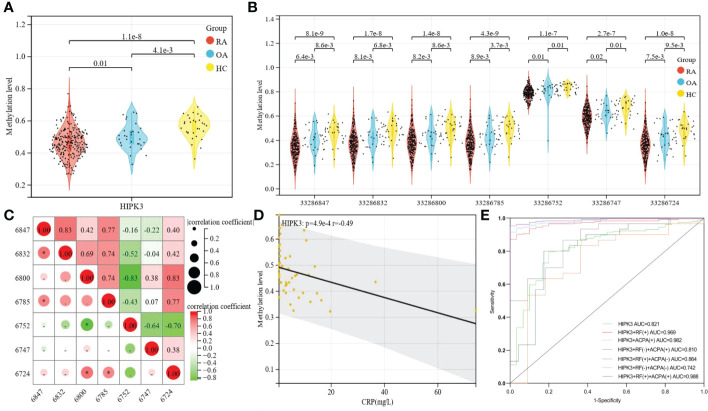
Differences in methylation level and correlation of HIPK3. **(A)**, Methylation level of HIPK3 between RA, OA patients and healthy controls; The result found that the methylation level of HIPK3 was down-regulated in patients with RA (P= 1.143×10^-8^). **(B)**, Methylation level of seven CpGs in different patients; Further analysis showed that HIPK3 has seven CpGs, including 33286847, 33286832, 33286800, 33286785, 33286752, 33286747, and 33286724; The difference analysis showed that the methylation levels of these CpGs in patients with RA were down-regulated, and the statistical difference of 33286785 is the most significant. **(C)**, Correlation analysis in different CpGs; The association analysis among seven CpGs showed that 6847 and 6832, 6800 and 6752 have strong correlation (|r|>0.8), and 6847 and 6785, 6832 and 6785, 6800 and 6785, 6724 and 6785, 6752 and 6724 have weak correlation. In general, there is a relatively close relationship between these CpGs. **(D)**, Correlation between HIPK3 and CRP in patients with 2.6 ≤ DAS28-CRP ≤ 3.2; In patients with 2.6 ≤ DAS28-CRP ≤ 3.2, there was a stronger correlation between HIPK3 and CRP (r= -0.49, p= 0.00049), indicating that HIPK3 has a higher clinical value in predicting CRP levels in these patients. **(E)** Multi-factor joint ROC analysis of HIPK3 combined with RF and ACPA; In multi-factor joint analysis, the results showed that compare with RF/ACPA, HIPK3 has more valuable clinical diagnostic significance in patients with RF (-)/ACPA (-) (AUC=0.742 vs 0.023). In addition, in patients with RF (+)/ACPA (+), adding the detection of HIPK3 methylation level was helpful to improve the diagnosis of RA (AUC=0.988), and HIPK3 could assist RF/ACPA as a new clinical index for the diagnosis of RA. *: P<0.05.

Using the Kruskal-Wallis rank sum test to calculate the P value between RA, OA and healthy groups. The whole blood methylation study found that HIPK3 (cg05501357) was down-regulated in patients with RA (P= 1.143×10^-8^; **Figure 1A**). Further analysis showed that HIPK3 has seven CpGs, including 33286847, 33286832, 33286800, 33286785, 33286752, 33286747, and 33286724. Using the Kruskal-Wallis rank sum test to calculate the P values between RA, OA and healthy groups. The difference analysis showed that the methylation levels of these CpGs in patients with RA were down-regulated (**Figure 1B**). The internal distribution can be perfectly shown by scatter plot, fitting curve and matrix diagram. Spearman’s rank correlation analysis was used to assess the association between two groups of data, and Based on the network data matrix, the correlation values between CpGs are analyzed and displayed visually by using the matrix correlation analysis and visualization function of Sangerbox (19) tool. An association analysis among all CpGs showed that 6847 and 6832, 6800 and 6752 have strong correlation (|r|>0.8), and 6847 and 6785, 6832 and 6785, 6800 and 6785, 6724 and 6785, 6752 and 6724 have weak correlation. In general, there is a relatively close relationship between these CpGs (**Figure 1C**). In the correlation analysis between the methylation level of HIPK3 and clinical indicators, it was found that HIPK3 was negatively correlated with CRP (r= -0.16, p= 0.01), and its seven CpGs also showed the same trend (**Table 1**). After that, the grouping analysis of RA patients showed that there was a stronger correlation between HIPK3 and CRP (r= -0.49, p= 0.00049) in patients with 2.6 ≤ DAS28-CRP ≤ 3.2, indicating that HIPK3 has a higher clinical value in predicting CRP levels in these patients (**Figure 1D**). In addition, the receiver operating characteristic curve (ROC) analysis of HIPK3 and the seven CpGs showed AUC scores were of 0.821, 0.823, 0.817, 0.818, 0.829, 0.798, 0.788, and 0.821 (**Supplement Figure 1A**). RF and ACPA are unable to play a role in diagnosing RA in patients with RF (–)/ACPA (-)(AUC=0.023; **Supplement Figure 2**). In this context, the detection of HIPK3 methylation can play a role in diagnosing RA to a certain extent. After joint analysis with RF (-)/ACPA (-) (AUC= 0.742; **Figure 1E**), which has better clinical diagnostic significance. Subsequently, multi-factor joint analysis showed that HIPK3 combined with RF and ACPA, and when the patient’s ACPA was negative, HIPK3 could assist RF as a new clinical index for the diagnosis of RA (AUC= 0.864; **Figure 1E**).

**Table 1:** Correlation analysis between the methylation level of different CpGs and clinical data.

Group	HIPK3	33286847	33286832	33286800	33286785	33286752	33286747	33286724
Age	0.05(0.42)	0.05(0.45)	0.05(0.46)	0.05(0.48)	0.05(0.46)	0.11(0.08)	0.10(0.14)	0.04(0.54)
BMI	0.02(0.78)	0.03(0.61)	0.02(0.73)	0.02(0.80)	0.02(0.72)	0.02(0.71)	0.03(0.70)	0.0022(0.97)
Course of disease	-0.04(0.58)	-0.04(0.51)	-0.04(0.55)	-0.04(0.52)	-0.04(0.55)	-0.03(0.66)	-0.004(0.95)	-0.04(0.54)
Age of onset	0.07(0.31)	0.07(0.29)	0.07(0.30)	0.07(0.30)	0.07(0.29)	0.09(0.16)	0.07(0.27)	0.06(0.33)
TJC	-0.02(0.74)	-0.02(0.76)	-0.03(0.69)	-0.02(0.76)	-0.02(0.73)	-0.01(0.83)	0.0092(0.89)	-0.02(0.77)
SJC	-0.02(0.77)	-0.03(0.70)	-0.01(0.86)	-0.01(0.85)	-0.02(0.81)	-0.04(0.50)	-0.0003(1)	-0.03(0.69)
VAS	-0.08(0.22)	-0.08(0.20)	-0.07(0.27)	-0.07(0.25)	-0.08(0.23)	-0.11(0.08)	-0.06(0.35)	-0.08(0.24)
RF	-0.02(0.75)	-0.0079(0.90)	-0.0053(0.93)	-0.005(0.94)	-0.01(0.85)	-0.06(0.39)	-0.06(0.39)	-0.02(0.74)
ACPA	0.04(0.57)	0.03(0.60)	0.04(0.55)	0.04(0.57)	0.04(0.55)	0.09(0.16)	0.02(0.81)	0,0.02(0.8)
ESR	-0.10(0.13)	-0.11(0.09)	-0.10(0.14)	-0.08(0.24)	-0.09(0.19)	-0.15(0.02)	-0.09(0.17)	-0.08(0.23)
CRP	-0.16(0.01)	-0.18(0.0061)	-0.16(0.02)	-0.14(0.04)	-0.15(0.02)	-0.18(0.0071)	-0.16(0.01)	-0.16(0.02)
DAS28-ESR	-0.08(0.20)	-0.09(0.17)	-0.08(0.22)	-0.07(0.27)	-0.08(0.23)	-0.11(0.08)	-0.05(0.41)	0.25 (-0.08)
DAS28-CRP	-0.09(0.16)	-0.10(0.14)	-0.09(0.17)	-0.08(0.21)	-0.09(0.19)	-0.11(0.10)	-0.06(0.35)	-0.09(0.18)

Correlation result format: r (p).

### Differences in methylation level and correlation of haplotype sites

**Figure 2:**
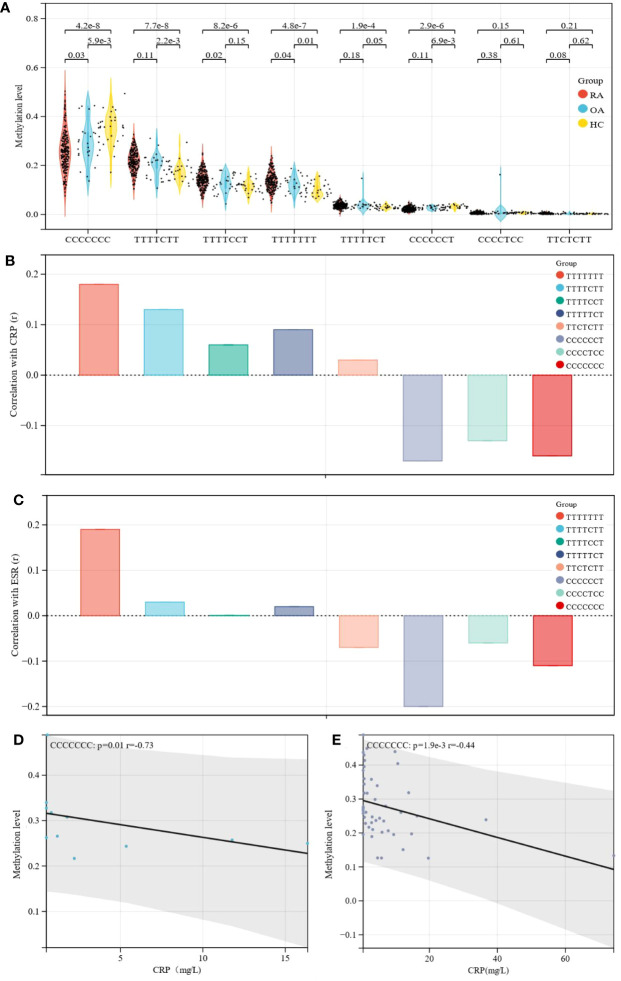
Differences in methylation level and correlation of haplotype sites. **(A)** Methylation level of different haplotype sites between RA, OA patients and healthy controls; Further analysis showed that HIPK3 has eight haplothpe sites, including TTTTTTT, TTTTCTT, TTTTCCT, TTTTTCT, TTCTCTT, CCCCCCT, CCCCTCC, CCCCCCC; The difference analysis showed that the methylation levels of these haplotype sites in patients with RA were different regulated, and among them, the methylation level of CCCCCCC in RA showed a downward trend, and the statistical difference was the most significant. **(B)** Correlation of haplotype sites with CRP were different compared with TTTTTTT; The correlation between different haplotype sites and CRP was analyzed, and the results were compared with TTTTTTT; Taking TTTTTTT as the standard, the trend of correlation between different haplotype sites and CRP was shown. **(C)** Correlation of haplotype sites with ESR was different compared with TTTTTTT; The correlation between different haplotype sites and ESR was analyzed, and the results were compared with TTTTTTT; Taking TTTTTTT as the standard, the trend of correlation between different haplotype sites and ESR was shown. **(D)** In patients with RF (-)/ACPA (-), CCCCCCC was negatively correlated with CRP; Further analysis of the correlation between haplotype sites and clinical indexes in different subgroups; The results showed that in patients with RF (-)/ACPA (-), correlation between CCCCCCC and CRP was the highest and the P value was statistically significant; CCCCCCC was negatively correlated with CRP level (r= -0.73, p= 0.01). **(E)** In patients with 2.6 ≤ DAS28-CRP ≤ 3.2, CCCCCCC were negatively correlated with CRP; Similarly, subgroup analysis also found that In patients with 2.6≤DAS28-CRP ≤ 3.2, there was also a statistically significant negative correlation trend between CCCCCCC and CRP (r= -0.44, p= 0.0019), and which was helpful to judge the change trend of clinical CRP level.

Similarly, the haplotype sites were also found to have significant differences in methylation in RA patients, OA patients and healthy controls (**Figure 2A**). Correlation analysis showed that the methylation level of CCCCCCT was negatively correlated with CRP (r= -0.17, p= 0.01) and ESR (r= -0.20, p= 0.0023), and CCCCCCC was negatively correlated with CRP (r= -0.16, p= 0.01) (**Table 2**). TTTTTTT indicated that there was no methylation in the HIPK3 gene, and in this state, the TTTTTTT level was positively correlated with ESR and CRP. Subsequently, when CCCCCCT, CCCCTCC, and CCCCCCC methylation occurred, the correlation between ESR and CRP also changed, and compared with TTTTTTT, the correlation was different (**Figures 2B, C**). Further association analysis showed that in patients with RF (-)/ACPA (-), CCCCCCC was negatively correlated with CRP level (r= -0.73, p= 0.01; **Figure 2D**). However, in patients with RF (+)/ACPA (+), CCCCCCT was negatively correlated with CRP (r= -0.17, p= 0.02) and ESR (r= -0.27, p= 0.00018) (**Supplement Figures 1B, C**). In patients with 2.6 ≤ DAS28-CRP ≤ 3.2, CCCCCCC (r= -0.44, p= 0.0019) (**Figure 2E**) and CCCCCCT (r= -0.42, p= 0.0033) (**Supplement Figure 1D**) were also negatively correlated with CRP.

**Table 2:** Correlation analysis between the methylation level of different haplotype sites and clinical data.

Group	TTTTTTT	TTTTCTT	TTTTCCT	TTTTTCT	TTCTCTT	CCCCCCT	CCCCTCC	CCCCCCC
Age	-0.07(0.28)	-0.09(0.16)	0.003(0.96)	0.03(0.68)	0.0083(0.90)	0.07(0.30)	-0.05(0.47)	0.02(0.78)
BMI	-0.04(0.55)	-0.04(0.51)	0.0087(0.89)	0.02(0.82)	-0.01(0.84)	0.10(0.12)	0.08(0.26)	-0.005(0.94)
Course of disease	0.05(0.48)	-0.01(0.83)	0.00089(0.99)	0.08(0.21)	-0.13(0.05)	0.00057(0.99)	-0.04(0.60)	-0.04(0.50)
Age of onset	-0.88(0.22)	-0.06(0.35)	-0.06(0.36)	0.01(0.82)	0.11(0.10)	0.04(0.52)	0.04(0.56)	0.05(0.44)
TJC	0.0092(0.89)	-0.01(0.87)	0.06(0.34)	-0.02(0.81)	0.10(0.14)	-0.02(0.72)	0.03(0.63)	-0.03(0.66)
SJC	0.05(0.45)	-0.01(0.83)	0.02(0.77)	-0.0019(0.98)	-0.05(0.49)	0.06(0.36)	-0.01(0.88)	-0.04(0.57)
VAS	0.12(0.06)	0.01(0.84)	0.07(0.27)	0.04(0.56)	0.09(0.19)	-0.17(0.01)	-0.02(0.73)	-0.07(0.26)
RF	0.08(0.21)	0.04(0.59)	-0.04(0.59)	-0.03(0.61)	-0.02(0.77)	-0.02(0.81)	-0.05(0.45)	-0.0093(0.89)
ACPA	-0.08(0.23)	0.01(0.84)	-0.02(0.73)	-0.06(0.38)	-0.11(0.11)	0.07(0.26)	-0.02(0.79)	0.0012(0.99)
ESR	0.19(0.0038)	0.03(0.69)	0.00071(0.99)	0.02(0.76)	-0.07(0.28)	-0.20(0.0023)	-0.06(0.42)	-0.11(0.11)
CRP	0.18(0.0061)	0.13(0.04)	0.06(0.37)	0.09(0.16)	0.03(0.68)	-0.17(0.01)	-0.13(0.06)	-0.16(0.01)
DAS28-ESR	0.12(0.06)	0.01(0.82)	0.06(0.33)	0.02(0.80)	0.02(0.72)	-0.13(0.05)	0.0066(0.92)	-0.09(0.16)
DAS28-CRP	0.10(0.13)	0.04(0.55)	0.08(0.25)	0.04(0.51)	0.06(0.37)	-0.11(0.08)	-0.02(0.78)	-0.09(0.16)

Correlation result format: r (p).

## Discussion

Rheumatoid arthritis (RA) is a systemic autoimmune disease caused by genetic and environmental factors. Owing to its heterogeneity and multiplicity, the etiology of RA remains largely unknown (20). Numerous studies have explored the attribution of the development of RA to genetic, infectious, and environmental factors (21–23). However, an increasing number of studies have shown that epigenetics plays an important role in the pathogenesis of RA, especially DNA methylation (24). Many studies have explored the pathogenesis of RA, but the interference of different epigenetic alterations in RA is not fully understood.

In addition to the comprehensive effects of environmental factors, genetic factors, and immune system disorders, inflammation plays an important role in the pathogenesis and pathological progression of RA (25). For example, interleukins (IL) can activate and regulate immune cells and mediate the activation of T and B cells, and their proliferation and differentiation play an important role in the inflammatory response. IL-1 and IL-6 were found to play a positive regulatory role and participate in the regulation mechanism of bone destruction in RA (26), while IL-23 is highly expressed in the serum of RA patients and is positively correlated with CRP and DAS-28 scores (27). Therefore, inflammatory reactions have received considerable attention in pathological studies of RA. CRP and ESR are associated with infection and tissue injury. They are non-specific markers of inflammation and directly participate in a series of acute and chronic inflammatory diseases, such as RA (28, 29). In this study, the methylation level of HIPK3 was negatively correlated with CRP (r= -0.16, p= 0.01). This confirmed the close relationship between the methylation levels of HIPK3 and CRP levels. In addition to CpGs, haplotype sites also serve as a medium for DNA methylation. Correlation analysis showed that the methylation level of CCCCCCT was negatively correlated with CRP (r= -0.17, p= 0.01) and ESR (r= -0.20, p= 0.0023), and CCCCCCC was negatively correlated with CRP (r= -0.16, p= 0.01).

Rheumatoid factor (RF) and anti-citrullinated protein antibodies (ACPA) are autoantibodies used in patients with RA. A study found that RF and ACPA have a synergistic effect in predicting developmental and bone erosion phenotypes (30), and their discovery has greatly promoted the early diagnosis and treatment of RA (31). Based on the above theoretical studies, we combined HIPK3 with RF (+), ACPA (+), RF (-)/ACPA (-) and RF (+)/ACPA (+) for ROC analysis. The results show that combined analysis can improve the clinical diagnostic value of HIPK3, especially in patients with RF (-)/ACPA (-) and RF (+)/ACPA (+).

The DAS-28 score is commonly used to evaluate disease activity in patients with RA; 2.6 ≤ DAS28-CRP ≤ 3.2 means that the patient was in a state of mild disease activity. Further research found that in patients with 2.6 ≤ DAS28-CRP ≤ 3.2, the methylation levels of HIPK3 (r= -0.49, p= 0.00049), CCCCCCC (r= -0.44, p= 0.0019), and CCCCCCT (r= -0.42, p= 0.0033) were negatively correlated with CRP, and the correlation is extremely high. Therefore, we can infer that in patients with RA, the methylation levels of HIPK3, CCCCCCC, and CCCCCCT are more sensitive to CRP and have greater clinical significance in predicting the trend of inflammatory reactions.

HIPK3 has been reported to be associated with tumorigenesis and apoptosis. Studies have found that circHIPK3 can act as an oncogene and autophagy regulator in lung cancer, and may be used as a prognostic marker and therapeutic target for lung cancer (32). In NSCLC tissues, low HIPK3 expression is associated with poor survival. Therefore, HIPK3 is considered a biomarker for predicting the survival of patients with NSCLC (15). However, the relationship between HIPK3 and RA has not been studied. In the current study, we found that the CpGs/haplotype site methylation level of HIPK3 was correlated with clinical indicators such as ESR, CRP, DAS28-CRP and RF/ACPA in RA patients. The correlation between HIPK3/haplotype sites and ESR and CRP, which are closely related to inflammation, was explained in detail in different characteristics of RA patients, and the clinical value of these sites in predicting the levels of ESR and CRP was also evaluated. Finally, the methylation level of HIPK3 in whole blood was closely related to the levels of ESR and CRP, and there was a significant negative correlation. In the ROC curve, through combinatorial analysis of HIPK3, RF (+) and ACPA (+), HIPK3 was found to have more clinical value in predicting the expression trend of inflammation. These results provide a basis and novel understanding for studying the relationship between inflammation and HIPK3 expression in RA.

## Data availability statement

The datasets presented in this study can be found in online repositories. The names of the repository/repositories and accession number(s) can be found below: SRA, PRJNA918808.

## Ethics statement

The studies involving human participants were reviewed and approved by Ethics Committee of Shanghai Guanghua Integrated Traditional Chinese and Western Medicine Hospital. The patients/participants provided their written informed consent to participate in this study. Written informed consent was obtained from the individual(s) for the publication of any potentially identifiable images or data included in this article.

## Author contributions

The original manuscript was completed by PJ and KW. SG, CC, RZ, YJ and YQ were accountable for the statistical analysis of data. LXX, JZ, LSX, SS and YS collected samples and helped in the statistical analysis. RW, YQ and DH contributed to the conception, design, and final approval of the submitted version. All authors reviewed and accepted the final version of the manuscript. All authors contributed to the article and approved the submitted version.
