# Supplementary material for: DNA methylation change of HIPK3 in Chinese rheumatoid arthritis and its effect on inflammation
Source: Front Immunol. 2023 Jan 10;13:1087279. doi: 10.3389/fimmu.2022.1087279 (PMC9872787; doi:10.3389/fimmu.2022.1087279)

**Supplementary Table 1** Basic information of the participants

| Group | HC | OA | RA | P value |
| --- | --- | --- | --- | --- |
| Sex (Female) | 30 | 30 | 235 | - |
| Number | 30 | 30 | 235 | - |
| Age (year) | 58.23 (1.653) | 61.23 (1.285) | 59.01 (0.689) | 0.460 |
| Height (cm) | 159.53 (0.928) | 158.30 (0.958) | 159.60 (0.308) | 0.376 |
| Weight (kg) | 60.87 (1.464) | 59.73 (1.424) | 59.24 (0.484) | 0.525 |
| Course of disease (year) | - | - | 11.82 (0.062) | - |
| Age of onset (year) | - | - | 47.20 (0.838) | - |
| TJC | - | - | 2.77 (0.263) | - |
| SJC | - | - | 1.53 (0.188) | - |
| VAS | - | - | 3.27 (0.139) | - |
| RF (+) | 0 | 0 | 203（86.38%） | - |
| ACPA (+) | 0 | 0 | 213（90.64%） | - |
| ESR (mm/h) | 10.42 (1.786) | 12.13 (1.384) | 23.62 (1.237) | 0.000 |
| CRP (mg/L) | 0.53 (0.034) | 2.99 (1.192) | 7.58 (0.840) | 0.000 |
| DAS28-ESR | - | - | 3.32 (0.088) | - |
| DAS28-CRP | - | - | 2.84 (0.080) | - |

**Supplementary Figure 1**

A, The ROC analysis of 7 CpGs. B, C, In patients with RF (+)/ACPA (+), the correlation of CCCCCCT with CRP (r=-0.17, p=0.02) and ESR (r=-0.27, p=0.00018). D, In patients with 2.6≤DAS28-CRP≤3.2, the correlation of CCCCCCT with CRP (r=-0.42, p=0.0033).


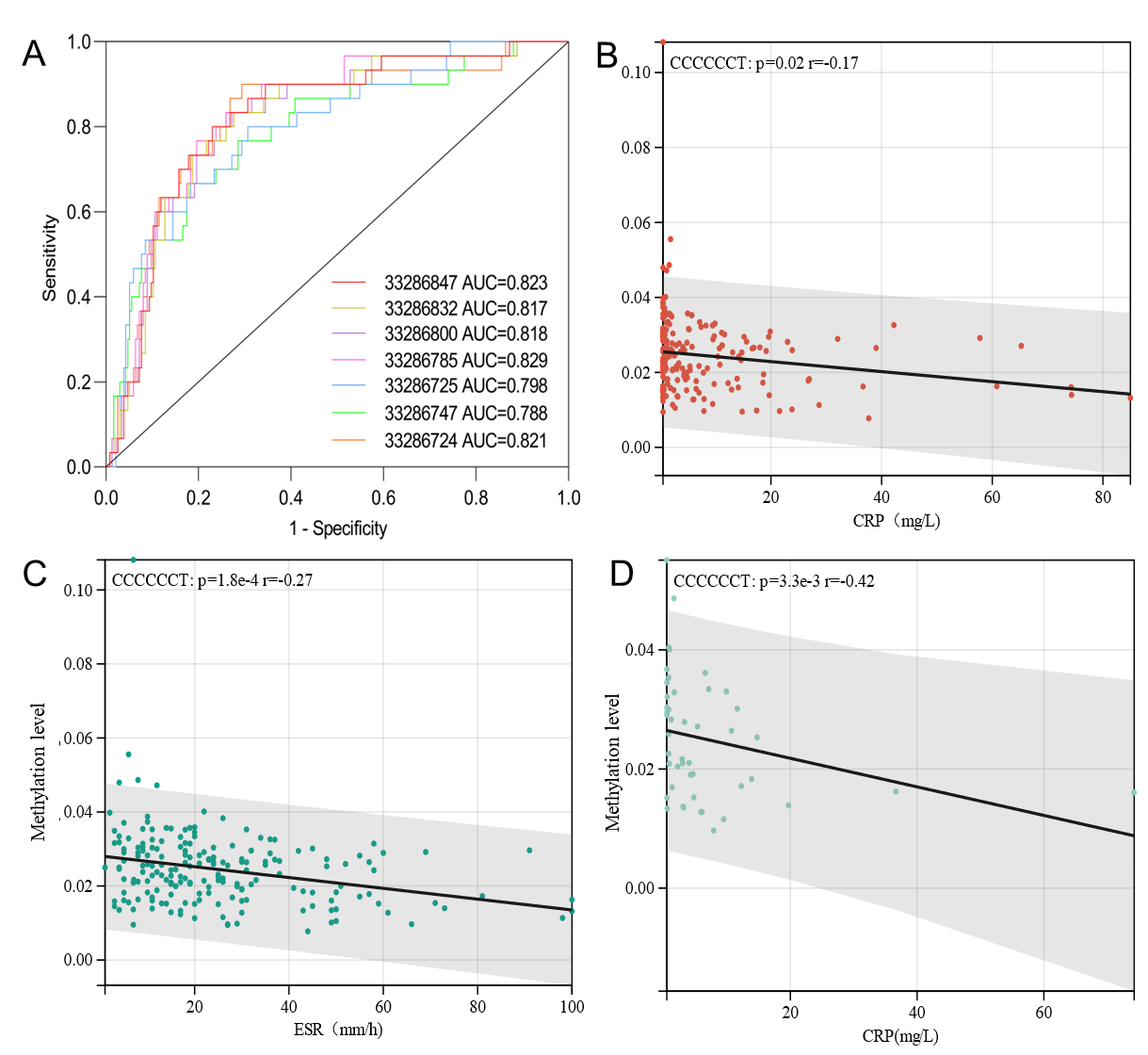


**Supplementary Figure 2**

The ROC analysis of RF (-)/ACPA (-), and the result showed that value of AUC is 0.023. This showed that RF (-) and ACPA (-) are unable to play a valuable role in diagnosing RA.


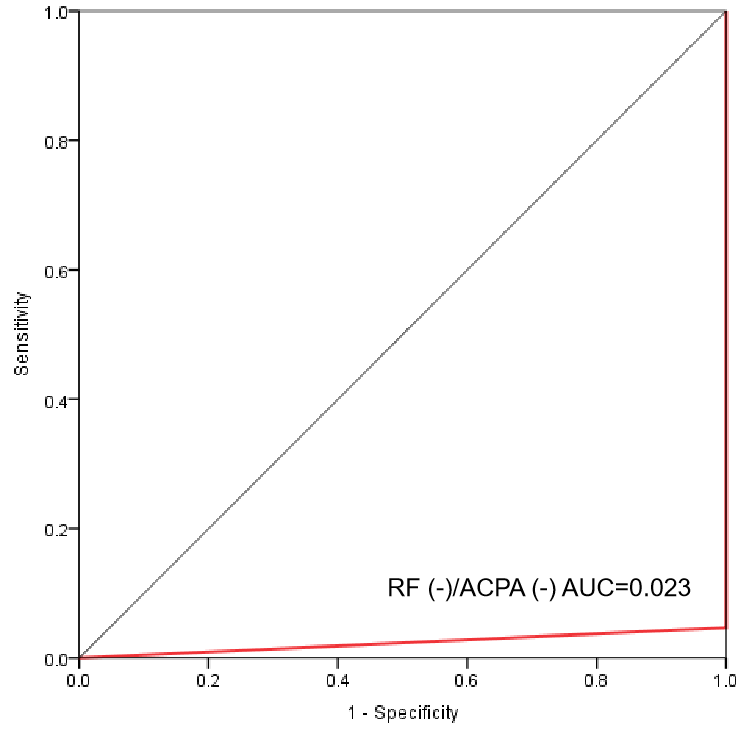

Supplement: Supplementary file 1 [file DataSheet_1.docx]
